# Current Status in the Therapy of Liver Diseases

**DOI:** 10.3390/ijms15057500

**Published:** 2014-04-30

**Authors:** Philipp Uhl, Gert Fricker, Uwe Haberkorn, Walter Mier

**Affiliations:** 1 Department of Nuclear Medicine, University Hospital Heidelberg, Im Neuenheimer Feld 400, 69120 Heidelberg, Germany; E-Mails: philipp.uhl@med.uni-heidelberg.de (P.U.); uwe.haberkorn@med.uni-heidelberg.de (U.H.); 2 Ruprecht-Karls-University, Institute of Pharmacy and Molecular Biotechnology, Im Neuenheimer Feld 329, 69120 Heidelberg, Germany; E-Mail: gert.fricker@uni-hd.de

**Keywords:** hepatic diseases, hepatitis, entry inhibitors, molecular mechanisms, liver cirrhosis, hepatocellular carcinoma

## Abstract

Hepatic diseases, like viral hepatitis, autoimmune hepatitis, hereditary hemochromatosis, non-alcoholic fatty liver disease (NAFLD) and Wilson’s disease, play an important role in the development of liver cirrhosis and, hence, hepatocellular carcinoma. In this review, the current treatment options and the molecular mechanisms of action of the drugs are summarized. Unfortunately, the treatment options for most of these hepatic diseases are limited. Since hepatitis B (HBV) and C (HCV) infections are the most common causes of liver cirrhosis and hepatocellular carcinoma, they are the focus of the development of new drugs. The current treatment of choice for HBV/HCV infection is an interferon-based combination therapy with oral antiviral drugs, like nucleos(t)ide analogues, which is associated with improving the therapeutic success and also preventing the development of resistances. Currently, two new protease inhibitors for HCV treatment are expected (deleobuvir, faldaprevir) and together with the promising drug, daclatasvir (NS5A-inhibitor, currently in clinical trials), adequate therapy is to be expected in due course (circumventing the requirement of interferon with its side-effects), while in contrast, efficient HBV therapeutics are still lacking. In this respect, entry inhibitors, like Myrcludex B, the lead substance of the first entry inhibitor for HBV/HDV (hepatitis D) infection, provide immense potential. The pharmacokinetics and the mechanism of action of Myrcludex B are described in detail.

## 1. Introduction

**Figure 1.**
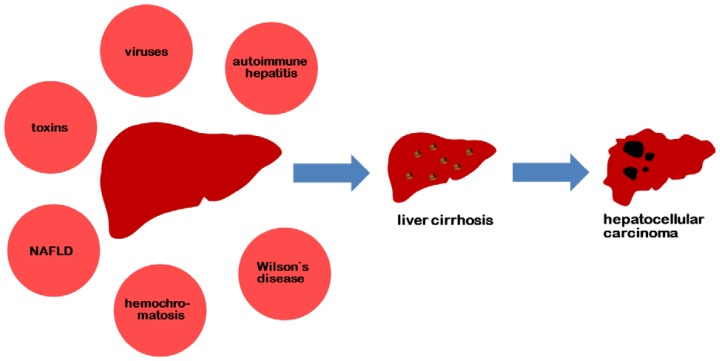
Risk factors for the development of liver cirrhosis with subsequent hepatocellular carcinoma.

Liver diseases, such as viral hepatitis, autoimmune hepatitis, hereditary hemochromatosis, non-alcoholic fatty liver disease (NAFLD) and Wilson’s disease, are associated with an increased risk for the development of liver cirrhosis and hepatocellular carcinoma. Additional risk factors are toxins, like alcohol and aflatoxin. The main risk factors for the development of liver cirrhosis are summarized in Figure 1.

While the prevalence of autoimmune hepatitis and metabolic disorders, like hemochromatosis and Wilson’s disease, is vanishingly low, more than 500 million people worldwide are persistently infected with the hepatitis B virus and/or hepatitis C virus [1]. Up to one million people die due to hepatitis B (HBV) infections and their consequences annually [2]. As the disease is only associated with nonspecific symptoms (the most common ones being malaise and fatigue) [3], the risk of developing liver cirrhosis or hepatocellular carcinoma is increased. An estimated 57% of the total hepatic cirrhosis and 78% of the primary hepatocellular carcinomas are the result of hepatitis B/hepatitis C (HCV) infections [2]. This highlights the importance for hepatitis infections to be diagnosed at an early stage to enable an optimal treatment.

Hepatitis infections can be caused by a wide range of viruses. Primary hepatotropic viruses (hepatitis A, B, C, D and E) are distinguished from other viruses, like Epstein-Barr virus or cytomegalovirus. Table 1 summarizes the differences of the family, genome, transmission path, incubation period and the possible immunoprophylaxis of the primary hepatotropic viruses.

In this review, the treatment options for the liver diseases mentioned above and their possible results, namely liver cirrhosis and hepatocellular carcinoma, are discussed.

## 2. Hepatitis

### 2.1. Hepatitis A

While hepatitis A infection is not associated with chronic liver disease in general, older and immunosuppressed people are susceptible to develop a fatal progression. Solid prophylaxis is possible, since hepatitis A is one of the best examples for vaccine-preventable infectious diseases in the world with several options for vaccination. The first effective vaccine against hepatitis A, Havrix™, was introduced in 1992 [4]. Nowadays, combination vaccines against hepatitis A/B are also available (Twinrix™ GlaxoSmithKline GmbH & Co. KG, 80700 München, Germany). These immunizations are indicated for people with a high work-related risk of infection (e.g., medical staff) and for people in regions with a high rate of hepatitis A infections, such as Western Africa.

### 2.2. Hepatitis B

Hepatitis B is one of the most threatening infectious diseases with an estimated 300 million chronic carriers worldwide [5]. In most cases, acute hepatitis B infection is associated with a spontaneous recovery, the reason for which therapy is not recommended [6]. Indeed, patients with life-threatening liver diseases and those with high levels of HBV replication and active or advanced liver diseases should be treated. Other patients should be monitored to ensure initiation of treatment when indicated (markers are HBV DNA levels and transaminases levels). Treatment of chronic hepatitis B is non-curative. It currently relies on seven registered drugs on the market that slow down disease progression [7]. The oldest and most common therapy consists of interferon-α-2a or peginterferon-α-2a. Interferon has antiviral, anti-proliferative and immunomodulatory effects. Consequently, the therapy can suppress HBV replication and also induce remission of liver diseases. Interferons are applied either in their native form or after conjugation with PEG (polyethylene glycol). PEGylation of interferon-α-2a leads to a slower enzymatic depletion and renal clearance. Peginterferon is also superior to unmodified interferon, because of its more convenient and predictable dosing schedule. Unfortunately, interferon therapy has also side-effects ranging from influenza-like illness, alopecia, leucopenia to thrombocytopenia. The most troublesome side-effect of interferon therapy is emotional lability, which manifests itself in anxiety, irritability and depression. Most of the side-effects cannot be predicted, but are reversible [8]. Nevertheless, interferon remains one of the first-line options for patients without cirrhosis [8]. In order to study the therapeutic success, patients receiving interferon therapy should have blood counts and liver panel monitored every four weeks and HBV DNA levels every 12 weeks. Unfortunately, all interferons have to be administered subcutaneously, whereas the alternative first-line option, the antiviral drugs, can be taken orally. Nowadays, five antiviral drugs are available: lamivudine (cytosine analogue), entecavir (guanosine analogue) and telbivudine (thymidine analogue), as well as the nucleotide-analogues, adefovir and tenofovir (both adenosine analogues), with a low prevalence of side-effects, but the risk of developing viral resistances and the disadvantage of a long-lasting treatment (the nucleos(t)ide analogues usually have to be administered for many years) [7]. Being prodrugs, these drugs are activated after their entry into the cell: nucleosides are triphosphorylated by cellular kinases, while nucleotides, which possess one phosphate group, have to be phosphorylated twice. In their respective triphosphate forms, they block the DNA-polymerase of the HBV and cause chain termination. Nucleos(t)ide therapy should be attended by liver panel monitoring every 12 weeks and HBV levels every 12–24 weeks. In addition, the serum creatinine and phosphorus blood levels should be tested every 12 weeks for patients receiving adefovir or tenofovir, because of their nephrotoxicity. In 2009, the American Association for the Study of Liver Diseases (AASLD) proclaimed key changes for first-line and second-line antiviral agents. In two double-blind randomized trials, tenofovir showed superiority when compared to adefovir as related to a higher number of patients with undetectable serum HBV DNA levels after 48 weeks of treatment and less occurrence of resistances up to 96 weeks of treatment [9]. Due to their advantages (see Table 2), tenofovir and entecavir are used as first-line oral antiviral drugs nowadays.

**Figure 2.**
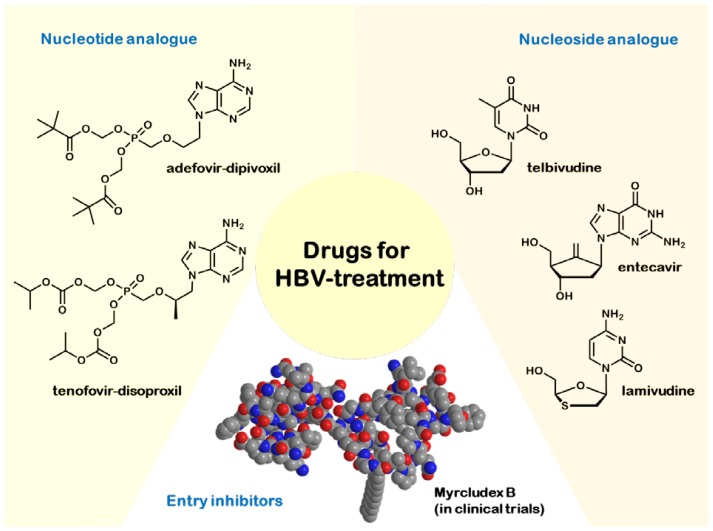
Drugs for HBV treatment.

The currently approved medications (shown in Figure 2) are non-curative, and the nucleos(t)ide analog-based treatment of chronic HBV infection frequently leads to the development of resistances. Consequently, alternative strategies and new drugs that target different steps of the HBV replication cycle are in demand to improve the treatment outcome [10]. For example, viral entry inhibition represents a potent therapeutic concept to combat viral infections both in the acute and the chronic phase. One approach could be the use of acylated peptides derived from the large HBV envelope protein. As shown in Table 1, HBV is a member of the Hepadnaviridae, the smallest enveloped DNA viruses that replicate by reverse transcription of a pregenomic RNA intermediate. During assembly of the nucleocapsid, HBV acquires three viral envelope proteins, termed large (L), middle (M) and small (S). These envelope proteins are encoded in one open reading frame and share the S-domain, which is required for membrane anchoring. In addition to the S-domain, M contains an *N*-terminal hydrophilic extension of 55 amino acids (preS2), while L is further extended by 107, 117 or 118 amino acids (genotype-dependent), termed preS1. The myristoylated preS1-domain of L plays the key role in HBV and hepatitis delta virus (HDV) infectivity by mediating attachment and specific receptor binding [11]. Petersen *et al.* [12] showed that hepadnavirus infection can be efficiently restrained by subcutaneous application of HBV envelope protein-derived lipopeptides *in vivo*. Schieck *et al.* [11] demonstrated that HBV virus hepatotropism is mediated through specific binding of the myristoylated *N*-terminal preS1-domain of the HBV L-protein to a hepatocyte specific, but at that time unknown, receptor. In 2013, Chen *et al.* [13] proclaimed a sodium taurocholate cotransporting peptide (NTCP) as a potential viral receptor by identifying a stretch of 10 amino acids in the NTCP transmembrane domain as the motive directly interacting with the preS1 peptide. In the future, there is the necessity to understand the interaction of NTCP with HBV envelope proteins and other cellular proteins. Schieck *et al.* [11] furthermore showed that Myrcludex B, the lead substance of the first entry inhibitor for HBV/HDV infection, is a very attractive potential drug, because of its exclusive targeting of susceptible cells. This makes subcutaneous application of low doses possible. Another beneficial effect is the remarkable serum stability of the peptide and a half-life time of about 16 h in mice, 10 h in rats and 13 h in beagles. Thus, therapeutic application may be required once every 1–3 days only. The peptide is currently in Phase 1/2 clinical trials. An important task in the future will be the development of a formulation of Myrcludex B, which enables its oral administration.

Beyond the NTCP receptor, other receptors that block HCV entry into the cell have been identified. Lupberger *et al.* [14] identified the epidermal growth factor receptor and ephrin receptor A2 as host cofactors for HCV entry, and in 2012, Sainz *et al.* [15] proclaimed the Niemann-PickC1-like 1 cholesterol absorption receptor (NPC1L1) as a new hepatitis C virus entry factor, since NPC1L1 expression is necessary for HCV infection. Ezetimibe, an Food and Drug Administration-(FDA) approved NPC1L1 antagonist, which is nowadays generally used as a lipid-lowering agent, potently blocks HCV uptake *in vitro* via a virion cholesterol-dependent step prior to virion-cell membrane fusion [16]. Together, these findings hold promise for the future that viral entry inhibitors could improve HCV treatment.

### 2.3. Hepatitis C

**Figure 3.**
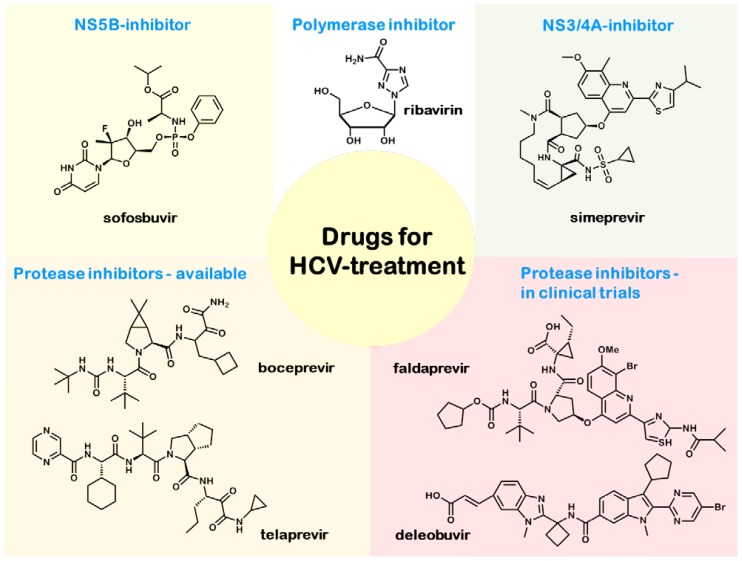
Drugs for HCV treatment.

Approximately 180 million people are chronically infected with the hepatitis C virus, and HCV infections are one of the most common reasons for liver cirrhosis and hepatocellular carcinoma. There are six genotypes of the hepatitis C virus that can be distinguished. Most important are the Genotypes 1–3 (Genotype 1 being the most common variant in the United States and Europe), which differ in their treatment options [6]. Acute hepatitis C infection remains asymptomatic in most cases, but is accompanied by the risk of chronic progression. Hence, it is generally treated with interferon α (PEGylated/non-PEGylated) [6], while the first-line treatment of chronic hepatitis C is a combination of peginterferon-α-2a/2b and the nucleoside analogue, ribavirin [17]. The dose of ribavirin administered and the duration of the treatment (48 weeks for Genotype 1, 24 weeks for Genotypes 2 and 3) depends on the genotype of the HCV virus. However, it is important to consider that the combination therapy shows more side-effects than the monotherapy. Because of its teratogenicity, ribavirin is contraindicated in pregnancy and the lactation period. It also shows hemolytic effects and a suppression of the myocardium. Since peginterferon plus ribavirin achieves sustained virological response in fewer than 50% of the patients with Genotype 1 infection, further therapies are required. The current therapeutic options for HCV Genotype 1 infections are shown in Figure 3 and include HCV protease inhibitors, like boceprevir and telaprevir, in addition to the interferon/ribavirin therapy. It was shown in patients with untreated chronic hepatitis C Genotype 1 infection that the addition of the protease inhibitor, boceprevir, to the standard treatment (peginterferon and ribavirin for 48 weeks) improves the response rate [18]. This triple therapy is not without side-effects, as it is associated with the selection of resistant viral variants, new adverse events and clinically relevant drug-drug interactions [19]. In 2013, two new drugs (sofosbuvir and simeprevir) received FDA approval. For this reason, the AASLD changed its recommendations for treatment-native patients with HCV Genotype 1 infection (see Table 3). Sofosbuvir (Sovaldi™ GILEAD Sciences GmbH, 82152 Martinsried, Germany) is a uridine nucleotide analogue and a selective inhibitor of the nonstructural protein 5B (NS5B), a viral protein found in the hepatitis C virus [20]. As a prodrug, sofosbuvir is activated intracellularly by phosphorylation. The second drug, simeprevir (Olysio™ Janssen Therapeutics, Division of Janssen Products, LP, Titusville NJ 08560), is a noncovalent inhibitor of the non-structural protease NS3/4A [21]. While sofosbuvir seems to be well-tolerated, unfortunately, simeprevir shows structural analogy to sulfonamides, and this part of the molecule is suspected to cause side-effects, like skin reactions. Other promising treatment options are currently under clinical investigation and are expected to be available in the second half of 2014. The novel treatment options include the second generation HCV protease inhibitor, faldaprevir, and deleobuvir, an HCV polymerase inhibitor. Both are currently being tested in Phase 3 clinical trials. A recent study (STARTVerso™ Boehringer Ingelheim, 55216 Ingelheim am Rhein, Germany) [22] investigated an interferon-based triple therapy (faldaprevir, peginterferon-α-2a and ribavirin) showing that faldaprevir is well-tolerated and achieves a significantly higher sustained virologic response. Another study investigated the effect of treatment duration, deleobuvir dosage and the absence or presence of ribavirin [23]. On the one hand, the absence of ribavirin could be an advantage, avoiding its severe side-effects, but on the other hand, as the study showed, the presence of ribavirin is a basic component for the success of these regimens. The latest drugs currently investigated in clinical trials are daclatasvir, an inhibitor of HCV replication with the non-structural protein 5A as the target (NS5A) and asunaprevir, a NS3 protease inhibitor. A Phase 2a study showed that the combination of these two drugs was well tolerated and achieved high response rates in genotype 1b HCV infections [24]. These findings give rise to the hope for an adequate therapy in the future.

### 2.4. Hepatitis D

Hepatitis D virus (HDV) is a unique RNA virus that requires a helper function provided by HBV for replication. Thus, HDV can only replicate in patients infected with HBV. The clinical course of hepatitis D infection is more severe than that of other hepatitis viruses, so that therapy should always be initiated. Unfortunately, there is no effective therapy for hepatitis D infection available nowadays. Interferon therapy is only associated with therapeutic success in about 30% of the treatments [6]. Moreover, there is no evidence yet that the combination therapy with ribavirin is superior to interferon monotherapy [6]. Hence, the only efficient possibility is the prevention of hepatitis B infection by vaccination, as discussed in a prior section.

### 2.5. Hepatitis E

Hepatitis E virus (HEV) most commonly occurs in Southeast Asia and parts of Africa, Central- and South America. Transmission of HEV occurs mainly by the fecal-oral route. Chronic progression is rare, and there was no specific therapy available; however, China’s State Food and Drug administration approved a vaccine (Hecolin™, Xiamen Innovax Biotech, Xiamen, China) in 2012 [25], and another vaccine is in clinical trials [26].

## 3. Others

### 3.1. Autoimmune Hepatitis

Autoimmune hepatitis is an inflammation of the liver of unknown cause. It reflects a complex interaction between triggering factors, autoantigens, genetic predispositions and immunoregulatory networks. Up to 40% of the patients suffering from autoimmune hepatitis develop liver cirrhosis [27]. The treatment of choice is a combination therapy of the corticosteroid, prednisone (immunosuppressive and anti-inflammatory effects), and the purine analogue, azathioprine. The addition of azathioprine allows the lowering of the dose of prednisone. The combination therapy is associated with a lower occurrence of corticosteroid-related side-effects, such as osteoporosis and hypertension [27]. Manns *et al*. [28] showed that the corticosteroid, budesonide, in combination with azathioprine, induces the remission of autoimmune hepatitis more effectively than the prednisone combination therapy with a low rate of steroid-specific side-effects. As a consequence, the budesonide therapy might become the first-line therapy once further data appear.

### 3.2. Hereditary Hemochromatosis

Hereditary hemochromatosis is an autosomal recessive disorder that leads to the accumulation of iron in the body as a result of mutations in a gene located on chromosome 6 (called the High Iron Fe (HFE) gene) [29]. Standard treatment consists of repeated phlebotomy to induce a negative iron balance and to remove excess iron stored in the tissues. Nutrition has only a low influence in hemochromatosis; vitamin C should be avoided, because it increases iron absorption, while the absorption of iron is decreased by black tea. Hutchinson *et al.* [30] showed that proton pump inhibitors can reduce the necessity to maintain phlebotomy by inhibiting the absorption of non-heme iron, so that treatments based on this mechanism may become available in the future.

### 3.3. Non-Alcoholic Fatty Liver Disease (NAFLD)

Non-alcoholic fatty liver disease (NAFLD), which is characterized by insulin resistance and necroinflammation with or without centrilobular fibrosis, increases the risk of developing diabetes and metabolic syndrome, resulting in liver cirrhosis and liver-related death [31,32]. This highlights the importance of adequate NAFLD-therapy. On the one hand, non-pharmacological therapy options, like weight reduction, physical activity and dietary changes, should be implemented. On the other hand, treatment of concurrent metabolic disorders, like obesity by statins or high blood pressure by antihypertensive agents, like beta-blockers, is necessary. Previous studies have investigated the possibility of treating NAFLD with pioglitazone, a thiazolidinedione that ameliorates insulin resistance and improves glucose and lipid metabolism in type 2 diabetes mellitus. Belfort *et al.* [31] showed that the administration of pioglitazone, in addition to diet, leads to metabolic and histologically visible improvement, but they also proclaimed that prolonged studies are necessary. Sanyal *et al.* [33] compared pioglitazone with vitamin E and placebo and showed that there was no benefit of pioglitazone over placebo for the improvement in histologic features. Certainly, pioglitazone showed benefits in reducing hepatic steatosis and lobular inflammation and an improvement in insulin resistance and liver-enzyme levels. Nevertheless, alternative therapy options with an improvement in histologic features have to be developed in the future.

### 3.4. Wilson’s Disease

Wilson’s disease is an autosomal recessive disorder of copper metabolism, which is caused by the absence or dysfunction of a copper transporting enzyme (P-type ATPase encoded on chromosome 13) [34]. It leads to a decrease in biliary copper excretion and finally to hepatic cirrhosis and neuronal degeneration. Therapeutic options include a diet to avoid copper-rich food (chocolate, mushrooms and Crustacea), as well as administration of the copper chelating agents, penicillamine (which is the drug of choice, but also shows severe side-effects) and trientine dihydrochloride, which leads to the excretion of copper in the urine [35]. Maintenance therapy of successfully treated symptomatic patients can also be accomplished by zinc. Zinc is a metallothionein inducer (a cysteine-rich protein that is an endogenous chelator of metals) with the ability to block the intestinal absorption of copper [36].

## 4. Liver Cirrhosis and Hepatocellular Carcinoma (HCC)

### 4.1. Liver Cirrhosis

Liver cirrhosis is a result of advanced liver diseases. Perz *et al.* [2] showed that in about 57% of the cases, liver cirrhosis was caused by HBV/HCV infection. Hence, prevention of liver cirrhosis and HBV/HCV infection is crucial, considering that about 78% of hepatocellular carcinoma (HCC) is attributable to HBV/HCV. The treatment of choice is the avoidance of boosting/triggering factors (alcohol, drugs, *etc*.) and the simultaneous therapy of the primary diseases (alcoholism, HBV/HCV infection).

### 4.2. Hepatocellular Carcinoma (HCC)

**Figure 4.**
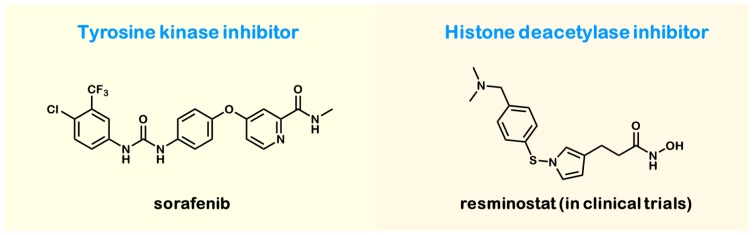
Drugs for hepatocellular carcinoma (HCC) treatment.

HCC is a major health problem, because no effective therapy is available. The main risk factors for the development of HCC are type 2 diabetes, alcoholism, toxins, such as aflatoxin, and, most particularly, the chronic HBV/HCV infections leading to liver cirrhosis, which is highly associated with HCC, as discussed above. Because of the poor response to cytostatic agents, the treatment of choice is surgical resection. Second-line options are percutaneous ethanol injection, radiofrequency ablation and liver transplantation. The only specific drug available is sorafenib (shown in Figure 4), a small molecule that inhibits tumor cell proliferation and angiogenesis. As a tyrosine kinase inhibitor, it blocks the autophosphorylation of the receptor tyrosine kinases like vascular endothelial growth factor (VEGFR), the platelet-derived growth factor receptor (PDGFR), and the proto-oncogenes c-Kit and RET. Studies [37] showed that in patients with advanced hepatocellular carcinoma, median survival and the time to radiologic progression were approximately three months longer for patients treated with sorafenib than for those given a placebo. A prospective possibility in HCC treatment could be resminostat, an oral available histone deacetylase inhibitor, because hypoacetylation of the histone protein H4 has been identified as a common early event in the pathogenesis of many human cancers, suggesting that increased histone deacetylase activity is a characteristic of malignant cells. Resminostat is currently being tested in Phase 2 studies as a monotherapy or in combination with sorafenib [38].

## 5. Conclusions

Within the numerous hepatic diseases treatment is mainly required for viral hepatitis infections (in particular hepatitis B and C) and hepatocellular carcinoma. In recent years new drugs for the treatment of hepatitis C such as sofosbuvir and simeprevir have been approved by the FDA. These new drugs enable improved therapy of hepatitis C. In contrast, the current treatment options for hepatitis B still lack the performance required. Consequently novel treatment options such as entry inhibitors are still demanded. Unfortunately, despite enormous efforts the treatment options for hepatic tumors, in particular hepatocellular carcinoma, are still insufficient.

## Figures and Tables

**Table 1.** Characteristics of hepatotropic viruses.

Characteristics	Hepatitis A virus	Hepatitis B virus	Hepatitis C virus	Hepatitis D virus	Hepatitis E virus
family	*Picornaviridae*	*Hepadnaviridae*	*Flaviviridae*	*unknown*	*Hepeviridae*
genome	single-stranded RNA	double-stranded DNA	single-stranded RNA	single-stranded RNA	single-stranded RNA
transmission route	fecal-oral	parenteral, sexual, perinatal	parenteral, sexual, perinatal	parenteral, sexual, perinatal	fecal-oral
incubation period	2–7 weeks	1–6 months	2–25 weeks	1–6 months	2–9 weeks
immunoprophylaxis	active, inactive	active, inactive	not available	active, inactive	not available

**Table 2.** Approved drugs for oral HBV treatment.

Characteristics	Lamivudine	Tenofovir	Adefovir	Entecavir	Telbivudine
year of approval	1999	2001	2002	2006	2007
resistance after five years	*ca*. 70%	not found yet	*ca*. 30%	*ca*. 1%	*ca*. 22%
medical assessment	well tolerated, main concern: resistance	less nephrotoxic than adefovir	nephrotoxic, less prone to resistance than lamivudine	well tolerated, more potent than lamivudine and adefovir	well tolerated, high potency, resistances appear after one year

**Table 3.** The American Association for the Study of Liver Diseases’ (AASLD) recommendations for the 12-week treatment of HCV genotype 1 infection.

Treatment-naive patients eligible to receive interferon	Treatment-naive patients ineligible to receive interferon
sofosbuvir (400 mg) daily	sofosbuvir (400 mg) daily
weight-based ribavirin daily	simeprevir (150 mg) daily
peginterferon on a weekly basis	with/without weight-based ribavirin
